# Updating movement estimates for American black ducks (*Anas rubripes*)

**DOI:** 10.7717/peerj.1787

**Published:** 2016-03-10

**Authors:** Orin J. Robinson, Conor P. McGowan, Patrick K. Devers

**Affiliations:** 1 School of Forestry and Wildlife Sciences, Auburn University, USA; 2 US Geological Survey, Alabama Cooperative Fish and Wildlife Research Unit, Auburn, AL, USA; 3 US Fish and Wildlife Service, Laurel, MD, USA

**Keywords:** American black duck, Banding data, Bayesian analysis, Migratory connectivity, Movement estimation

## Abstract

Understanding migratory connectivity for species of concern is of great importance if we are to implement management aimed at conserving them. New methods are improving our understanding of migration; however, banding (ringing) data is by far the most widely available and accessible movement data for researchers. Here, we use band recovery data for American black ducks (*Anas rubripes*) from 1951–2011 and analyze their movement among seven management regions using a hierarchical Bayesian framework. We showed that black ducks generally exhibit flyway fidelity, and that many black ducks, regardless of breeding region, stopover or overwinter on the Atlantic coast of the United States. We also show that a non-trivial portion of the continental black duck population either does not move at all or moves to the north during the fall migration (they typically move to the south). The results of this analysis will be used in a projection modeling context to evaluate how habitat or harvest management actions in one region would propagate throughout the continental population of black ducks. This analysis may provide a guide for future research and help inform management efforts for black ducks as well as other migratory species.

## Introduction

Effectively conserving migratory species requires knowledge of the demographic processes occurring at breeding, non-breeding areas, and the migratory pathways connecting these areas. Often, knowledge of connectivity between breeding and non-breeding areas is a limiting element for effective management and conservation (Faaborg et al., 2010). The demographic processes in one of these stages of a migratory species’ life cycle are also likely to affect the others (Marra, Hobson & Holmes, 1998; McGowan et al., 2011). Knowing where migratory species move and how they get there can also shed light on the metapopulation dynamics that may occur among distinct populations of these species, furthering our understanding of which ‘stocks’ within a population may be at higher risk of decline (Esler, 2000; Zimpfer & Conroy, 2006). Another goal of understanding migration is in identifying important stopover sites (Mehlman et al., 2005; Skagen, 2006). Understanding migratory pathways and the role of migration along them in shaping the demography of populations can allow conservation practitioners to make complicated decisions about how best to spend limited capital (Conroy, Stodola & Cooper, 2012; Desholm et al., 2014). Estimating connectivity between breeding grounds, stopover sites and wintering grounds can help managers understand how management enacted on one part of the annual cycle will interplay with other parts of the annual cycle.

Large strides have recently been made in understanding inter-seasonal migratory connectivity (Webster et al., 2002; Robinson et al., 2009), with new techniques currently being tested and implemented using stable isotopes (Hobson et al., 2014), genetic markers (Ruegg et al., 2014), geolocators (Catry et al., 2014) and mark-recapture modeling (Cohen et al., 2014). However, there are drawbacks to each of these approaches. Stable isotope analysis and genetic markers require rather expensive equipment, as there is some cost in developing the assays for the genetic marker analysis. Results from stable isotope studies may not be as accurate as basic probability models (Wunder et al., 2005). Provided one has the equipment and the assays for genetic analysis, it would cost roughly $10 (USD) per individual bird (Ruegg et al., 2014). This is still less expensive than the $25 (USD) per sample for stable isotope analysis and the $150 per unit for geolocators (Hobson et al., 2014). In addition to their great expense, geolocators have also been shown to have a negative effect on survival and reproduction (Scandolara et al., 2014) and require recapturing the individual sometime after initial application to recover the location data. Modeling migratory connectivity via analysis of marked individuals requires capturing an individual at least once, then seeing that same individual and/or capturing it again. For some species, this has proven very difficult (e.g., Pied Flycatcher (*Ficedula hypoleuca*); Webster et al., 2002). Mathematical models, such as those used in modeling movement from marked individuals, are frequently seen as black-boxes (Addison et al., 2013) and not as transparent as simply attaching a geolocator to an individual and determining where it went once the bird is recaptured and the data are recovered.

While banding (ringing) data has its issues, it is still the most widely used and accessible way to estimate migratory connectivity. One may access more than 70 million banding and 5 million encounter records from the North American Bird Banding Laboratory (BBL) and over 10 million encounter records from EURING (www.pwrc.usgs.gov/bbl; www.euring.org; Cohen et al., 2014). Individuals may also receive information about birds they may have observed in the field and begin building a database through sites such as www.bandedbirds.org. Banding data has proven useful in many contexts, including movement estimation for many different groups including doves (Collier et al., 2012), waterfowl (Lavretsky et al., 2014), shorebirds (Gill, Handel & Ruthrauff, 2013), raptors (Goodrich et al., 2012) and songbirds (Suomala, Morris & Babbitt, 2012).

American black ducks (*Anas rubripes*, here after black ducks) are a harvested, international migratory waterfowl species in eastern North America (Loncore et al., 2000). There were significant long term declines in black duck populations between the 1960s and 1990s according to winter count data and breeding season monitoring (Devers & Collins, 2011). Despite an extended period of restrictive harvest regulations (Francis, Sauer & Serie, 1998) the black duck population is still below the population goal identified in the North American Waterfowl Management Plan (NAWMP) (Devers & Collins, 2011). The Black Duck Joint Venture (BDJV) was created in 1989 under NAWMP to develop monitoring and research programs that will aid in the continental black duck population achieving the NAWMP goal.

It has been hypothesized that density dependent factors restrict population growth in black duck populations and that habitat management (increases, improvements, etc.) may be a key component of growing black duck populations and reaching the prescribed NAWMP population goal (Conroy, Miller & Hines, 2002; Devers & Collins, 2011). However, deciding how much habitat to preserve and where to acquire or restore that habitat at the continental scale to best benefit black duck populations is first and foremost limited by understanding how habitat management actions in any portion of the annual life cycle will affect the overall population (Devers et al., 2010; Devers & Collins, 2011). To aid in management decision making, the NAWMP has called for building and parameterizing full annual cycle (FAC) population models for black ducks, as well as many other waterfowl species (Anderson et al., 2007).

Following the NAWMP, the framework for a FAC population model has been created (Robinson et al., 2016) that fits the management units outlined by the BDJV. The first step in parameterizing a large scale optimal habitat management model for migratory species is understanding how wintering and breeding region populations are connected to each other (Hostetler, Sillett & Marra, 2015). There have been studies conducted to evaluate black duck movement in the past; however, these studies used data from birds banded between 1971–1994 (Conroy, Fonnesbeck & Zimpfer, 2005) and between 1965–1998 (Zimpfer & Conroy, 2006). These studies also used different regions in their movement estimations than the regions drawn by the BDJV. Here, we have updated the estimation of movement probabilities for black ducks using band recovery data. We estimate the number of birds banded in each of the seven regions defined by the BDJV and the subsequent movement of those birds among the regions. Our study adds 27 additional years of data to the most recent analysis for estimating cross seasonal, continental scale movements (Zimpfer & Conroy, 2006). Furthermore, we did not limit our data to only those black ducks that were banded in the traditional breeding areas and did not discard data for those birds migrating northward or not migrating at all as previous studies have done (Zimpfer & Conroy, 2006).

## Materials and Methods

**Figure 1:**
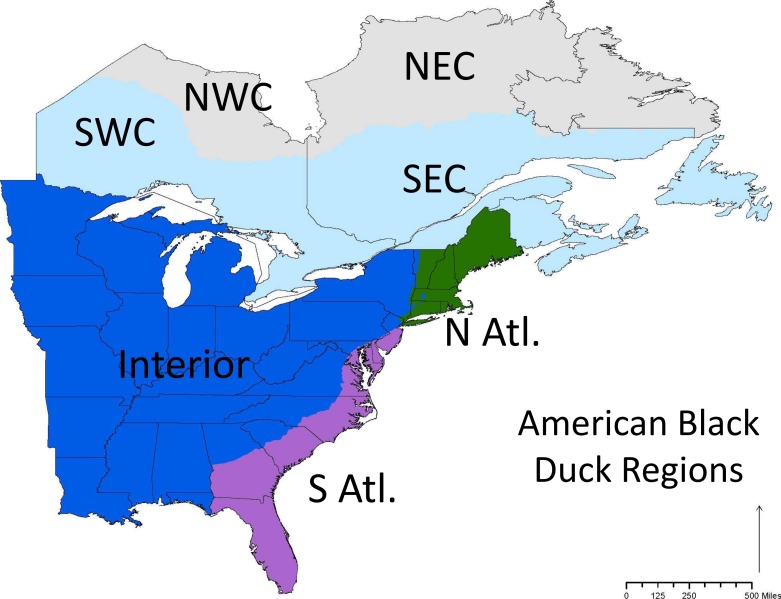
The conservation regions set by the Black Duck Joint Venture. Note that some regions are named relative to the known range of black ducks so that NW Canada is the part of Canada that is in the northwest part of the ABDU range, and not the true northwest portion of Canada.

We obtained more than 50,000 band recoveries of black ducks banded in the United States and Canada from 1951–2011 from the BDJV. We only included birds that were banded during the preseason (1 July–30 Sept.) and recovered during the hunting season. Birds that were recovered in the hunting season immediately after they were banded (i.e., they were banded and recovered in the same year) and within the region in which they were banded were removed from the analysis. The removal of these birds was to ensure that the individuals at least had an opportunity to move between the banding and recovery events. Since migration occurs primarily between October and February, any birds recovered in those months that was banded in the banding period immediately preceding migration (that same year) were not included in our analysis. We split the data into seven regions (e.g., four breeding regions and three wintering regions; Fig. 1) and black ducks banded in all of the seven regions were included in the study. The regional boundaries were set by the BDJV habitat integration working group as these are the regions that will be used for future simulation modeling to inform continental habitat management strategies (Devers et al., 2010; Devers & Collins, 2011; Robinson et al., 2016).

Using a hierarchical Bayesian approach, we calculated the probability (*π*) that an individual moved from region *i* to region *j*, was recovered (e.g., shot by a hunter) and reported as

\begin{eqnarray*}{\pi }_{ij}={\Psi }_{ij}\lambda h \end{eqnarray*}

following Conroy, Fonnesbeck & Zimpfer (2005), where Ψ is the movement probability, *λ* is the reporting probability and *h* isthe harvest rate in the region. Ψ was informed by an uninformative multinomial distribution (Dirichlet distribution) where the movement probabilities were constrained to sum to one. *λ* was drawn from a beta distribution with *α* and *β* parameters calculated so that the distributions for the reporting probability for each band type had a mean equal to the reporting probability of each band type provided by the BDJV (P Devers, 2015, unpublished data). This differs slightly form previous analyses where *λ* was assumed to be constant. We had multiple band types, each with different reporting probabilities. Previous studies have recommended including these data to analyses such as ours if it is available (Conroy, Fonnesbeck & Zimpfer, 2005). We constrained *h* to be constant with respect to each region. As *h* and *λ* are confounded, this assumption must be made so that Ψ is identifiable. Therefore, a continental harvest rate was drawn from a beta distribution with *α* = 2 and *β* = 19 to produce a distribution with a mean of 0.055 (value for mean taken from Conroy, Miller & Hines, 2002). The data (*D*_*ij*_; the number of birds recovered in region *j* that were banded in region *i*) was then modeled as binomial distribution

\begin{eqnarray*}{D}_{ij}\sim Bin({\pi }_{ij},N) \end{eqnarray*}

where *N* is the total number of birds banded. Using OpenBUGS (Thomas & O’Hara, 2007), values for Ψ_*ij*_ were then sampled via MCMC from its posterior distribution to create a new distribution of estimates for Ψ_*ij*_ influenced by the prior distribution and the data. This analysis was performed for each band type, and the results weighted by the proportion of the data contributed for each band type. The model was sampled to convergence according to convergence diagnostics using the package CODA (Plummer et al., 2006) in program R (R Core Team, 2013).

**Table 1:** Mean (standard deviation) movement probabilities for black ducks. Regions in which individuals were banded are on the left side of the table and regions in which individuals were encountered are across the top.

	NWC	NEC	SWC	SEC	N Atl.	S Atl.	Int.
NWC	0.033(0.056)	0.018(0.013)	0.021(0.025)	0.036(0.016)	0.031(0.018)	0.4(0.048)	0.461(0.067)
NEC	0.009(0.02)	0.012(0.003)	0.006(0.006)	0.391(0.019)	0.287(0.015)	0.21(0.017)	0.08(0.008)
SWC	0.007(0.02)	0.005(0.015)	0.002(0.001)	0.049(0.007)	0.032(0.03)	0.325(0.026)	0.581(0.05)
SEC	0.006(0.012)	0.020(0.019)	0.034(0.004)	0.135(0.007)	0.227(0.017)	0.354(0.027)	0.225(0.021)
N Atl.	0.003(0.002)	0.025(0.02)	0.016(0.005)	0.155(0.012)	0.294(0.032)	0.342(0.025)	0.164(0.014)
S Atl.	0.009(0.004)	0.032(0.044)	0.07(0.016)	0.186(0.068)	0.044(0.04)	0.551(0.108)	0.108(0.021)
Int.	0.004(0.003)	0.004(0.003)	0.1(0.03)	0.052(0.009)	0.028(0.005)	0.311(0.017)	0.502(0.027)

## Results

Once the data were constrained to the banding and recovery periods described above, we were left with 14,624 black ducks recovered in the United States and Canada from 1951–2011. Ducks that were banded in NW, SW, and NE Canada rarely stayed in that region during the migration period (3.3% for NW Canada; 0.2 % for SW Canada; 1.2% for NE Canada; Table 1). Of the ducks banded in SE Canada, 13.5% stayed in the region; however, 39% of the ducks banded in NE Canada moved to SE Canada. For ducks banded in the S Atlantic and Interior regions, more than 50% stayed within the region during the migration period, while 29.4% of the ducks banded in the N Atlantic stayed in that region. For NW and SW Canada, about 90% of birds banded in those regions moved to one of the wintering regions. For ducks banded in NE Canada, 58% moved to one of the wintering regions, and for those banded in SE Canada, 80% moved to a wintering region. For ducks banded in the United States, more than 15% in each region moved north during the fall migration period.

## Discussion

Here, we have extended the previous efforts of Conroy, Fonnesbeck & Zimpfer (2005) and Zimpfer & Conroy (2006) to estimate movement of black ducks. Our simple update to previous methods (incorporating multiple band types and their different reporting probabilities) has allowed us to include an additional 27 years of data. The reporting of different band types has changed over the years; from bands that required the person who made the recovery to mail the band and location to the bird banding laboratory, to those that require an easily accessible online form to be filled out. Occasionally, there are “reward” bands placed on birds that offer a reward to the person who reports the band (these are usually assumed to be reported at 100% and typically used to estimate reporting probabilities of non-reward bands). All of these band types have different reporting probabilities (Williams, Nichols & Conroy, 2002). In order to consider long-term movement probabilities, one must include them all rather than assuming one reporting probability or choosing a subset of data from bands with only one reporting probability. This was recognized by Conroy, Fonnesbeck & Zimpfer (2005), who suggested the use of such data when possible. Being able to use long-term banding data for movement analysis allows us to detect trends in the movement data. For example, climate change has been shown to have an effect on waterfowl abundance and on timing of migration (Sorenson et al., 1998; Guillemain et al., 2015). Using a method such as ours, with multiple band types and allowing for birds that do not migrate out of their region, one could potentially determine how the movement (or lack of movement) of black ducks among regions has changed over time in response to changes in habitat availability, climate, or other aspects of environmental change.

Movement analyses may help influence habitat management by suggesting the most important regions used by the species of interest. Our results show that many black ducks use the Atlantic coast during the winter months. We estimate that 33–63% of black ducks, regardless of breeding region, move into or remain in either the N Atlantic or S Atlantic regions. This corroborates a recent meta-analysis suggesting the importance of the Atlantic coast as vital black duck wintering habitat (Ringelman et al., 2015). Further, Aagaard et al. (2015) showed that habitat was the most important factor among a suite of variables for predicting winter black duck abundance in the Atlantic flyway. These studies, along with our movement analysis strongly suggest that habitat improvements along the Atlantic coast of the United States would greatly benefit the continental black duck population.

We found that black ducks generally exhibit flyway fidelity (e.g., individuals banded in the western breeding regions tended to over winter in western regions, but see above) similar to Addy (1953), Conroy, Fonnesbeck & Zimpfer (2005), Zimpfer & Conroy (2006) and Lavretsky et al. (2014). There may be some error or bias in the results. For example, some northward movements that we observed may be post breeding/fledging movement prior to migrating and settling into an over wintering home range. Using only birds banded in the preseason and recovered during the hunting season tried to limit the potential temporally caused biases in our results. Using large regional boundary definitions also reduces the effect of location reporting errors. Some of these northward movements may also be local scale movements of birds that, for example, were banded in northern Maine and recovered in New Brunswick some years later. If this is a concern, one could adjust the analysis to account for distance moved as well as movement across the boundaries of the regions. The regional boundaries also reflect the region definitions used in a larger population simulation modeling effort that will evaluate continental scale habitat management strategies (Devers et al., 2010; Devers & Collins, 2011; Robinson et al., 2016). The objective of that annual cycle modeling effort is to identify which region and part of the annual cycle should habitat management be focused on to best benefit black duck populations (Robinson et al., 2016). This analysis can provide a way to prioritize management efforts by acting as a component of a larger modeling effort (Conroy, Stodola & Cooper, 2012). The results can be used in the recent projection modeling context to evaluate how habitat or harvest management actions in one region would propagate throughout the continental population of black ducks (Robinson et al., 2016). The results of these analyses serve as the first step in parameterizing projection models and developing a framework for making continental scale habitat management decisions to support black duck populations and to fit the needs of the BDJV. Our results also demonstrate the potential utility of banding data for estimating interseasonal connectivity of migratory species, with much less expense than genetic, isotope or geolocator techniques.

## Supplemental Information

10.7717/peerj.1787/supp-1 Supplemental Information 1 Banding Data from BDJV

10.7717/peerj.1787/supp-2 Supplemental Information 2 Example BUGS code for analysis

## Additional Information and Declarations

### Data Availability

The following information was supplied regarding data availability: Data has been provided as Supplemental Information.
