# Supplementary material for: Updating movement estimates for American black ducks (Anas rubripes)
Source: PeerJ. 2016 Mar 10;4:e1787. doi: 10.7717/peerj.1787 (PMC4793334; doi:10.7717/peerj.1787)
Supplement: Supplemental Information 2 [file peerj-04-1787-s002.docx]

model

{

#prior probabilities of birds with each band type moving to one of the 7 regions. Each calculated prob. is #a Dirichlet prior

for(i in 1:7){

mband1[i]~dgamma(1,1)

moveband1[i]<-mband1[i]/(sum(mband1[]))

}

for(i in 1:7){

mband2[i]~dgamma(1,1)

moveband2[i]<-mband2[i]/(sum(mband2[]))

}

for(i in 1:7){

mband3[i]~dgamma(1,1)

moveband3[i]<-mband3[i]/(sum(mband3[]))

}

for(i in 1:7){

mband4[i]~dgamma(1,1)

moveband4[i]<-mband4[i]/(sum(mband4[]))

}

DP1~dbeta(99,1) #Detection probability for different bands modeled as beta dist

DP2~dbeta(16.5,34) # Means- DP1=1, DP2=0.32, DP3=0.5, DP4= 0.71

DP3~dbeta(20,20)

DP4~dbeta(35.5,15)

HR~dbeta(2,19) # Harvest Rates used by Conroy et al 2002

#Below is the model describing the data

# move_rec_B1_B2 = prob of a bird with band type 1 moving to B1

#moveband1 = prob of a bird with band type 1 moving from a given region (B1, B2, B3, W1 etc.; from #prior calculation above)

# DP1 = detection probability of band 1

# HR = harvest rate (from constant above)

move_rec_B1_B1<-moveband1[1]*DP1*HR # data = prob moved*detection probabilities *harvest (with different bands)

move_rec_B2_B1<-moveband2[1]*DP2*HR

move_rec_B3_B1<-moveband3[1]*DP3*HR

move_rec_B4_B1<-moveband4[1]*DP4*HR

move_rec_B1_B2<-moveband1[2] *DP1*HR

move_rec_B2_B2<-moveband2[2]*DP2*HR

move_rec_B3_B2<-moveband3[2]*DP3*HR

move_rec_B4_B2<-moveband4[2]*DP4*HR

move_rec_B1_B3<-moveband1[3]*DP1*HR

move_rec_B2_B3<-moveband2[3]*DP2*HR

move_rec_B3_B3<-moveband3[3]*DP3*HR

move_rec_B4_B3<-moveband4[3]*DP4*HR

move_rec_B1_B4<-moveband1[4]*DP1*HR

move_rec_B2_B4<-moveband2[4]*DP2*HR

move_rec_B3_B4<-moveband3[4]*DP3*HR

move_rec_B4_B4<-moveband4[4]*DP4*HR

move_rec_B1_W1<-moveband1[5]*DP1*HR

move_rec_B2_W1<-moveband2[5]*DP2*HR

move_rec_B3_W1<-moveband3[5]*DP3*HR

move_rec_B4_W1<-moveband4[5]*DP4*HR

move_rec_B1_W2<-moveband1[6]*DP1*HR

move_rec_B2_W2<-moveband2[6]*DP2*HR

move_rec_B3_W2<-moveband3[6]*DP3*HR

move_rec_B4_W2<-moveband4[6]*DP4*HR

move_rec_B1_W3<-moveband1[7]*DP1*HR

move_rec_B2_W3<-moveband2[7]*DP2*HR

move_rec_B3_W3<-moveband3[7]*DP3*HR

move_rec_B4_W3<-moveband4[7]*DP4*HR

N_B1~dpois(est_B1) #Estimated number of birds that could have moved for each band type

N_B2~dpois(est_B2)

N_B3~dpois(est_B3)

N_B4~dpois(est_B4)

NE<-N_B1+N_B2+N_B3+N_B4 #Estimated total number of birds that could have moved

Det_B1_B1~dbin(move_rec_B1_B1,N_B1) #Fitting the data to the model

Det_B1_B2~dbin(move_rec_B2_B1,N_B2)

Det_B1_B3~dbin(move_rec_B3_B1,N_B3)

Det_B1_B4~dbin(move_rec_B4_B1,N_B4)

Det_B2_B1~dbin(move_rec_B1_B2,N_B1)

Det_B2_B2~dbin(move_rec_B2_B2,N_B2)

Det_B2_B3~dbin(move_rec_B3_B2,N_B3)

Det_B2_B4~dbin(move_rec_B4_B2,N_B4)

Det_B3_B1~dbin(move_rec_B1_B3,N_B1)

Det_B3_B2~dbin(move_rec_B2_B3,N_B2)

Det_B3_B3~dbin(move_rec_B3_B3,N_B3)

Det_B3_B4~dbin(move_rec_B4_B3,N_B4)

Det_B4_B1~dbin(move_rec_B1_B4,N_B1)

Det_B4_B2~dbin(move_rec_B2_B4,N_B2)

Det_B4_B3~dbin(move_rec_B3_B4,N_B3)

Det_B4_B4~dbin(move_rec_B4_B4,N_B4)

Det_W1_B1~dbin(move_rec_B1_W1,N_B1)

Det_W1_B2~dbin(move_rec_B2_W1,N_B2)

Det_W1_B3~dbin(move_rec_B3_W1,N_B3)

Det_W1_B4~dbin(move_rec_B4_W1,N_B4)

Det_W2_B1~dbin(move_rec_B1_W2,N_B1)

Det_W2_B2~dbin(move_rec_B2_W2,N_B2)

Det_W2_B3~dbin(move_rec_B3_W2,N_B3)

Det_W2_B4~dbin(move_rec_B4_W2,N_B4)

Det_W3_B1~dbin(move_rec_B1_W3,N_B1)

Det_W3_B2~dbin(move_rec_B2_W3,N_B2)

Det_W3_B3~dbin(move_rec_B3_W3,N_B3)

Det_W3_B4~dbin(move_rec_B4_W3,N_B4)

#Calculating number of birds that moved to each region.

# B1=Number that moved to B1

# Weighting the number of birds with each type of band, and adding those that moved to each region

B1<-(moveband1[1]*(N_B1/NE))+(moveband2[1]*(N_B2/NE))+(moveband3[1]*(N_B3/NE))+(moveband4[1]*(N_B4/NE))

B2<-(moveband1[2]*(N_B1/NE))+(moveband2[2]*(N_B2/NE))+(moveband3[2]*(N_B3/NE))+(moveband4[2]*(N_B4/NE))

B3<-(moveband1[3]*(N_B1/NE))+(moveband2[3]*(N_B2/NE))+(moveband3[3]*(N_B3/NE))+(moveband4[3]*(N_B4/NE))

B4<-(moveband1[4]*(N_B1/NE))+(moveband2[4]*(N_B2/NE))+(moveband3[4]*(N_B3/NE))+(moveband4[4]*(N_B4/NE))

W1<-(moveband1[5]*(N_B1/NE))+(moveband2[5]*(N_B2/NE))+(moveband3[5]*(N_B3/NE))+(moveband4[5]*(N_B4/NE))

W2<-(moveband1[6]*(N_B1/NE))+(moveband2[6]*(N_B2/NE))+(moveband3[6]*(N_B3/NE))+(moveband4[6]*(N_B4/NE))

W3<-(moveband1[7]*(N_B1/NE))+(moveband2[7]*(N_B2/NE))+(moveband3[7]*(N_B3/NE))+(moveband4[7]*(N_B4/NE))

}

#DATA

#First 2 lines are unused

#Det_B1_B3= number of birds recovered in B1 with band type 3 (B3)

list(a_B1=1,b_B1=999,a_B2=1,b_B2=999,a_B3=1,b_B3=999,a_B4=1.25,b_B4=48.75,a_W1=0.6,b_W1=49.4, a_W2=21.5,b_W2=28.5, a_W3=26.55,b_W3=23.45,

Det_B1_B1=0, Det_B1_B2=0, Det_B1_B3=0,Det_B1_B4=0,

Det_B2_B1=0, Det_B2_B2=0, Det_B2_B3=0,Det_B2_B4=0,

Det_B3_B1=0, Det_B3_B2=0, Det_B3_B3=0,Det_B3_B4=0,

Det_B4_B1=0, Det_B4_B2=2, Det_B4_B3=0,Det_B4_B4=0,

Det_W1_B1=0, Det_W1_B2=1, Det_W1_B3=0,Det_W1_B4=0,

Det_W2_B1=0, Det_W2_B2=32, Det_W2_B3=2,Det_W2_B4=0,

Det_W3_B1=0, Det_W3_B2=34, Det_W3_B3=7,Det_W3_B4=1,

est_B1=0,est_B2=215.625,est_B3=18,est_B4=1.408)
